# Krüppel‐like factors family in health and disease

**DOI:** 10.1002/mco2.723

**Published:** 2024-09-10

**Authors:** Tingwen Xiang, Chuan Yang, Zihan Deng, Dong Sun, Fei Luo, Yueqi Chen

**Affiliations:** ^1^ Department of Orthopedics Southwest Hospital Third Military Medical University (Army Medical University) Chongqing China; ^2^ Department of Biomedical Materials Science Third Military Medical University (Army Medical University) Chongqing China; ^3^ Department of Orthopedics Chinese PLA 76th Army Corps Hospital Xining China

**Keywords:** bone destruction diseases, bone homeostasis, energy metabolism, epigenetic modification, Krüppel‐like factors (KLFs), systemic diseases

## Abstract

Krüppel‐like factors (KLFs) are a family of basic transcription factors with three conserved Cys2/His2 zinc finger domains located in their C‐terminal regions. It is acknowledged that KLFs exert complicated effects on cell proliferation, differentiation, survival, and responses to stimuli. Dysregulation of KLFs is associated with a range of diseases including cardiovascular disorders, metabolic diseases, autoimmune conditions, cancer, and neurodegenerative diseases. Their multidimensional roles in modulating critical pathways underscore the significance in both physiological and pathological contexts. Recent research also emphasizes their crucial involvement and complex interplay in the skeletal system. Despite the substantial progress in understanding KLFs and their roles in various cellular processes, several research gaps remain. Here, we elucidated the multifaceted capabilities of KLFs on body health and diseases via various compliable signaling pathways. The associations between KLFs and cellular energy metabolism and epigenetic modification during bone reconstruction have also been summarized. This review helps us better understand the coupling effects and their pivotal functions in multiple systems and detailed mechanisms of bone remodeling and develop potential therapeutic strategies for the clinical treatment of pathological diseases by targeting the KLF family.

## Graphical abstract

KLFs are pivotal factors in regulating essential processes across eight major systems: nervous, cardiovascular, respiratory, digestive, endocrine, urinary, reproductive, and musculoskeletal systems. This graphic illustrated how KLFs influenced cellular functions and disease mechanisms within each system, highlighting their diverse roles in maintaining health and their involvement in various pathologies.

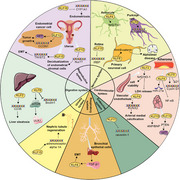

## 1 INTRODUCTION

Krüppel‐like factors (KLFs) are a family of transcription factors characterized by the presence of three C2H2‐type zinc finger motifs at their C‐terminus, which are responsible for binding to specific DNA sequences, thereby regulating the transcription of target genes. Since its identification as the mammalian homolog gene of the Krüppel in Drosophila melanogaster in 1993, the KLF family has continued to grow with the identification of more KLF members.^1^ These factors are involved in the regulation of gene expression related to cellular differentiation, proliferation, and apoptosis. Recent research has revealed that the influence of KLFs extends beyond individual cellular processes to encompass broader systemic functions, which are proved to widely participate in regulating various biological processes and be integral to maintaining homeostasis in various tissues and organs, influencing both health and disease states.^2^ Certain KLFs support cardiovascular health via modulating endothelial cell function and heart muscle adaptation, with disruptions potentially leading to atherosclerosis and heart failure.^3^, ^4^ Within the immune system, KLFs impact inflammation and immune cell functions, and also play essential roles in tissue repair and cancer progression.^5^, ^6^, ^7^ Meanwhile, its dysregulation can be associated with neurodegenerative diseases.^8^

Recently, emerging evidence has highlighted their crucial involvement in the skeletal system, where they contribute to bone homeostasis between bone formation and resorption. Bone is a dynamic tissue that is constantly going through remodeling, in which organic and inorganic components are formed by osteoblasts (OBs) and absorbed by osteoclasts (OCs).^9^ OCs derive from hematopoietic progenitors in response to macrophage‐colony stimulating factor (M‐CSF) and receptor activator of nuclear factor‐kappaB (NF‐κB) ligand (RANKL),^10^, ^11^ which are a kind of large and multinucleated cells with the ability to degrade bone matrix by secreting H^+^, Cl^−^, cathepsin K and matrix metalloproteinases (MMPs) into the resorption lacuna.^12^, ^13^ The differentiation of OCs requires the concerted activation of transcription factors including c‐fos, c‐jun, nuclear factor of activated T cell c1 (NFATc1), and NF‐κB.^14^, ^15^ Individually, OBs are bone‐forming cells with mesenchymal origin that secrete proteins and deposit minerals to reconstruct the bone matrix. The differentiation of OBs requires an orchestrated series of events to modulate activities of transcription factors including Runt‐related transcription factor 2 (Runx2), Osterix, and β‐catenin.^16^, ^17^ The coordination of stage‐specific transcription factors is essential for the maintenance of bone homeostasis, orchestrating the OB–OC coupling processes. Aberrant expression or function of many of these transcription factors cause disrupted balanced activities of OCs and OBs, associated with the breaking of bone remodeling and the occurrence of bone‐related diseases.^18^, ^19^ Among these transcription factors, KLFs, a group of zinc finger transcription factors, have been proven to affect the differentiation of OBs and OCs and participate in their coupling cross‐talk, efficaciously maintaining bone homeostasis.^20^, ^21^ The effect of KLFs on bone homeostasis is not only mediated through conventional pathways but also by energy metabolism alteration and epigenetic modification. Various KLF family members are expressed in bone‐related cells and contribute to the regulation of bone cell proliferation, differentiation, and function, either working together synergistically or in an antagonistic manner.^22^

The transition from understanding KLFs’ systemic roles to their specific actions in bone underscores the importance of these factors in maintaining skeletal homeostasis and addressing bone‐related disorders. By bridging systemic functions with localized bone effects, KLFs emerge as key players in orchestrating both general physiological processes and specialized skeletal functions. While much has been learned about KLFs in general, their precise mechanisms of action and interactions with other transcription factors in various developmental stages and disease conditions are not fully understood. In this review, we summarized the pivotal roles of KLFs across the major systems and concentrated on KLF‐related regulatory mechanisms in pathophysiological bone homeostasis, discussed the promising role of KLFs in health and diseases, and aimed to provide a theoretical foundation for the establishment of therapeutic strategies for treating bone destruction diseases by targeting KLFs.

## 2 THE BIOLOGICAL CHARACTERISTICS AND FUNCTIONS OF THE KLF FAMILY

**FIGURE 1:**
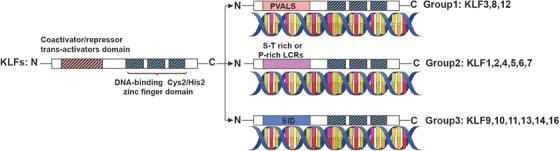
Protein structure and transcriptional coregulator of KLF family members. KLF protein has three conserved DNA‐binding Cys2/His2 zinc finger domains in C‐terminal regions and various protein interaction domains in N‐terminal regions. KLF family is grouped according to their shared domain architectures: (1) Group 1: KLF 3, 8, and 12 contain a PVDLS domain and bind to CtBP; (2) Group 2: KLF1, 2, 4, 5, 6, and 7 contain S/T‐rich or P‐rich LCRs; (3) Group 3: KLF9, 10, 11, 13, 14, and 16 contain SID.

There are 18 identified members in the KLF family, which function as transcription factors characterized by the three conserved DNA‐binding Cys2/His2 zinc finger domains located in their C‐terminal ends. All KLFs share the highly conserved C‐terminal domains to combine with similar DNA sequences such as CACCC‐, GC‐, or GT‐box elements.^23^ By contrast, their N‐terminal regions display high diversity, containing various protein interaction domains, which mediate the interactions among KLFs and transcriptional regulatory proteins including transcriptional coregulators and other chromatin remodeling proteins.^24^ The members of the KLF family can be divided into three groups according to their shared domain architectures.^25^, ^26^ Group1: consists of KLF proteins 3, 8, and 12, which contain a PVDLS domain and act as transcriptional repressors by interacting with C‐terminal binding protein (CtBP). Group2: KLF proteins include KLF1, 2, 4, 5, 6, and 7 with serine/threonine (S/T)‐rich or proline (P)‐rich low complexity regions (LCRs), serving as transcriptional activators. Group 3: KLF9, 10, 11, 13, 14, and 16 share a conserved Sin3a interacting domain (SID), which mediates their activity of transcriptional repressors.^27^ KLF15 and KLF17 have not been assigned to any group because of limited knowledge about their protein interaction motifs, and the KLF18 gene is believed to be a pseudogene^28^ (Figure 1).

The effects of KLFs on the transcriptional process have been elucidated, along with the mechanisms by which they interact with transcriptional regulatory proteins. For instance, members of group 2 may bind to coregulators that have acetyltransferase activity, such as cAMP response element binding protein (CBP), p300, and p300/CBP‐associated factor (P/CAF)^29^, ^30^, ^31^, ^32^, ^33^; whereas members of group 1 may bind to CtBP^34^, ^35^, ^36^ and members of group 3 may bind to Sin3A.^37^ KLFs interact with CtBPs to repress transcription by recruiting proteins that affect chromatin remodeling, including histone deacetylases (HDACs) and histone methyltransferases (HMT).^38^ CtBPs can also bind and restrain HAT coactivators such as p300/CBP, as well as recruit other repressors to induce transcriptional gene silencing.^39^ Histone acetyltransferases (HATs) such as CBP, p300, and P/CAF induce acetylation of KLFs stimulating their transcriptional activity.^30^, ^40^ Alternatively, HATs can directly acetylate histones, resulting in chromatin remodeling and initiation of transcription for target genes. Sin3A has been proposed as a potential scaffold for the assembly of a complex comprising HDAC, Mad, Ume6, MeCP2, N‐CoR, silencing mediator of retinoid and thyroid receptor, which can exert inhibitory effects on the expression of target genes.^41^, ^42^ Besides the transcriptional regulatory proteins, posttranslational modifications such as acetylation, phosphorylation, ubiquitination, and sumoylation, are also important in regulating the functions of KLFs via promoting the combination with DNA, increasing the transcription activity of KLFs, enhancing nuclear translocation or leading the degradation of KLF and so on.^28^

KLFs participate in many cellular physiological activities including cell proliferation, differentiation, and apoptosis, and often with context‐dependent functions depending on cell type and differentiation stage.^43^, ^44^ Specially, various studies have also proved that KLFs exhibited a close connection with bone dynamic activity.^45^ Understanding the extensive roles of KLFs in these systems is essential for elucidating their contributions to health and diseases.

## 3 MULTIFACETED ROLES OF KLFs IN HEALTH AND DISEASE

KLFs are transcription factors crucial for regulating various biological processes across health and disease spectrums. They maintain cellular integrity and systemic homeostasis, influencing physiological activity such as metabolic processes, and immune responses. In disease contexts, KLFs are pivotal in managing metabolic disorders, cancer progression, inflammatory conditions, and so on, participating in multiple pathological and physiological changes.

### 3.1 Nervous system

KLFs play a crucial role in the development, function, and regeneration of the nervous system, responsible for regulating physiological processes such as neuronal differentiation, synaptic formation, and neuroprotective mechanisms. Specifically, KLF4 is known to be involved in the self‐renewal and differentiation of neural stem cells within the nervous system,^46^, ^47^ as well as participate in nerve regeneration after injury, which facilitates remyelination and functional recovery after spinal cord injury via inducing astrocyte reprogramming.^48^ KLF4 has been also identified as an effective inhibitor of axon growth in retinal ganglion cells (RGCs) and functions to repress axon and dendrite initiation and elongation by hippocampal neurons in vitro.^49^ Additionally, KLF4 demonstrates a neuroprotective role through the regulation of the Nrf2/Trx1 pathway.^50^

Both KLF6 and KLF7 have been shown to promote neurite growth.^49^ A detailed investigation into KLF7 within the nervous system certified that knocking out KLF7 leads to impairments in axon growth and pathfinding in various areas such as the olfactory system, retina, and brain.^51^ Moreover, the capacity of KLF7 to boost the expression of Trk neurotrophin receptors might be crucial for the survival of RGCs following optic nerve injury.^52^ Therefore, KLF7 could not only accelerate neurite growth but also potentially enhance the responsiveness of neurons to trophic factors, ultimately improving neuroprotection postinjury.

KLF8 has been shown to regulate the expression of met and p53 crucial for the development of granule and purkinje cells,^53^ as well as activate the Wnt/β‐catenin signaling pathway, potentially impacting the progression of Alzheimer's disease.^54^ KLF9 contributes to late‐phase neuronal maturation in the developing dentate gyrus and during adult hippocampal neurogenesis,^55^ which exerts various functions across different neuronal types.^8^ Furthermore, KLF15 acts as a transcriptional repressor of the rhodopsin and interphotoreceptor retinoid‐binding protein (IRBP) promoters, as well as involved in restraining photoreceptor‐specific gene expression in nonphotoreceptor cells.^56^

### 3.2 Cardiovascular system

KLF2 has the potential to enhance endothelial nitric oxide synthase uncoupling through the Nrf2/HO‐1 pathway in cases of endothelial injury, leading to improved cell viability, decreased lactate dehydrogenase (LDH) release, and a reduction in the oxidative stress response.^57^ In endothelial cells, KLF2 suppresses prothrombotic factors like plasminogen activator inhibitor 1 and tissue factor, while simultaneously promoting the upregulation of the antithrombotic factor thrombomodulin (TM) in inflammatory conditions.^4^, ^58^

KLF4 has been discovered to be expressed in various vascular cell types and contributes to the progression of vascular diseases by regulating the transcription of multiple genes through interactions with different partner proteins. Specially, KLF4 inhibits the expression of smooth muscle cell differentiation markers via associating with serum response factor, HDACs, and binding to p53. Furthermore, KLF4 collaborates with Runx2 to reinforce arterial medial calcification.^59^

KLF5 has a variety of effects, such as promoting vascular smooth muscle cell proliferation, lipid deposition in the vessels, and inflammation, all of which substantially participate in atherosclerosis.^60^, ^61^ KLF5 could activate Cyclin D1 and suppress p21,^62^ and its interaction with poly‐ADP‐ribose‐polymerase‐1 obstructs the proapoptotic effect,^63^ making a contribution to rescuing cells from undergoing apoptosis in the early phase and accelerating cell growth in the late phase to stimulate vascular remodeling in case of injury. Besides, KLF14 is also implicated in the regulation of diseases and pathological processes related to atherosclerosis.^3^ On the one hand, research has demonstrated that KLF14 plays a protective role by inhibiting lipoprotein lipase mediated by miR‐27a, as well as diminishing the secretion of proinflammatory cytokines and lipid accumulation.^64^ On the other hand, KLF14 suppresses the NF‐κB signaling pathway by impeding the production of p65, thereby decreasing leukocyte adhesion to activated endothelial cells.^65^

KLF13 is primarily expressed in the heart involved in the transcription network required for heart development.^66^ which functions as a pivotal protective element that prevents cardiomyocytes from DNA damage and death.^67^ Consistently, inhibition of KLF10 results in the alteration of Pten/Akt signaling to reduce cardiomyocyte apoptosis as well as enhance higher proliferation, playing a cardioprotective role in ischemic heart disease.^68^ In patients with heart failure, KLF15 participates in the adapting response to heart pressure overload via regulating the expression of atrial natriuretic factor (ANP) and B‐type natriuretic peptide (BNP).^69^ Moreover, through interactions with factors including myocardin, TGF‐β, myocyte enhancer factor 2, and GATA‐binding protein 4,^70^ KLF15 is able to suppress cardiac fibrosis and hypertrophy, ultimately consummating heart function upon myocardial remodeling processes triggered by mechanical or metabolic factors.^71^

### 3.3 Respiratory system

KLF4 performs distinct capabilities in the management of profibrotic mediators in various lung cell types. KLF4 in platelet‐derived growth factor receptor (PDGFR)‐β^+^‐derived cells is of vital significance in the overload of myofibroblasts and extracellular matrix (ECM) generation and accumulation. Conversely, KLF4 in SMA^+^ cells exhibits a protective effect, and especially, downregulation of its expression could account for an increase in lung myofibroblasts.^72^ Furthermore, KLF4 in myeloid cells including macrophages and polymorphonuclear neutrophils serves as a crucial regulator of the early proinflammatory immune response. The stimulation of both cell types with *S. pneumoniae* dramatically boosts KLF4 expression, thus promoting a proinflammatory phenotype.^73^

KLF6, as a downstream factor of LOX‐1/TGF‐β1 signaling pathway, is implicated in the pathological advancement of epithelial–mesenchymal transition (EMT)‐mediated pulmonary fibrosis with diabetes.^74^ Consistently, KLF15 also has the potential to counteract endoplasmic reticulum stress and inhibit excessive proliferation, migration, and ECM accumulation in lung fibroblast.^75^ Besides, KLF7 has been uncovered to be highly expressed in lung adenocarcinoma tissues and associated with unsatisfied clinical outcomes.^76^ KLF9 deficiency has been revealed to decrease the level of inflammatory factors and downregulate GSDMD expression, thereby alleviating lung injury and inflammatory responses.^77^

### 3.4 Digestive system

KLF2 primarily exerts vasoprotective functions through activating target genes including endothelial nitric oxide synthase, TM, and c‐type natriuretic peptide in liver sinusoid endothelial cells.^78^ Nevertheless, KLF2 also upregulates CD36 expression via a binding site on its proximal promoter region, which partially contributes to liver steatosis.^79^ KLF6 could mitigate the level of cellular oxidative stress and promote the responsibility for harmful stimuli. Further, with the modulation of Beclin1 transcription and activation of the mTOR/ULK1 pathway, KLF6 restrains excessive autophagy overactivation in order to safeguard the liver against ischemia/reperfusion injury.^80^

Except for the parenchymal organs, the impacts of KLFs across the digestive tract deserve attention. It has been manifested that KLF4 ameliorates intestinal permeability, substantially enhances intestinal tight junction, and alleviates endotoxemia via repressing NF‐κB transcription activity.^81^ KLF4, KLF5, KLF6, and KLF8 have been implicated in gastric carcinogenesis. Both KLF4 and KLF6 perform tumor suppressors.^82^, ^83^ KLF5 and KLF8 appear to promote the proliferation, invasion, and metastasis of human gastric carcinoma cells.^84^, ^85^ In colorectal tumors, KLF4, KLF6 and KLF9 have been identified to suppress colorectal carcinogenesis.^86^, ^87^ KLF4 restrains the expression of genes related to cell‐cycle progression including *CCND1* and *ODC*.^88^ In contrast, KLF5 functions as colorectal carcinogenesis promoter.^89^

### 3.5 Endocrine system

In the endocrine system, KLFs influence the biosynthesis, secretion, and function of various hormones by regulating gene expression, which in turn has a substantial impact on endocrine activity. KLF11 specifically controls the expression of the insulin genes by interacting with the insulin promoter and has been identified as a causal factor for maturity‐onset diabetes of the young 7 (MODY7).^90^ Besides, KLF11 functions as a dominant inhibitor of the caveolin‐1 gene in response to cholesterol signals.^91^ KLF14 has also been implicated in the pathophysiological progress of metabolic diseases, such as obesity, insulin resistance, and T2D.^92^

KLFs also play a crucial role in the differentiation of adipocytes and the metabolism of fatty acids. KLF3 orchestrates lipid metabolism via improving fatty acid β‐oxidation (FAO).^93^ KLF15 in adipose tissue modulates insulin exudation and resistance through diminishing stearoyl‐CoA desaturase 1 and oxidative stress,^94^ and it also has the capacity to shift fuel between glucose and fatty acids under different energy statuses in brown adipose tissue, which might enhance FAO accompanied by the promotion of the expression of Acox1 and Fatp1 while attenuating glucose oxidation.^95^

### 3.6 Urinary system

KLFs are extensively expressed in tissues including kidney, bladder, and urethra, where they play crucial roles in the development, maintenance, and repair of the urinary system. These factors control genes associated with cell proliferation, differentiation, apoptosis, and inflammatory response, impacting both normal physiological conditions and diseases. KLF15 directly binds enhancers of regenerative genes such as adrenoreceptor alpha 1A to facilitate their expression in Xenopus laevis, the downregulation of which pharmacologically hinders nephric tubule regeneration, while the activation plays recuperating roles.^96^

### 3.7 Reproductive system

Uterine and ovarian pathologies have been revealed to be both closely associated with the dysregulation of KLFs. Interestingly, higher levels of KLF12 were found in human endometrial cancer tissues compared with normal endometrium. The upregulation of KLF12 boosts cell proliferation and migration, as well as reins apoptosis through the activation of AKT signaling and promotion of CCND1 expression level, ultimately contributing to tumor growth.^97^

**FIGURE 2:**
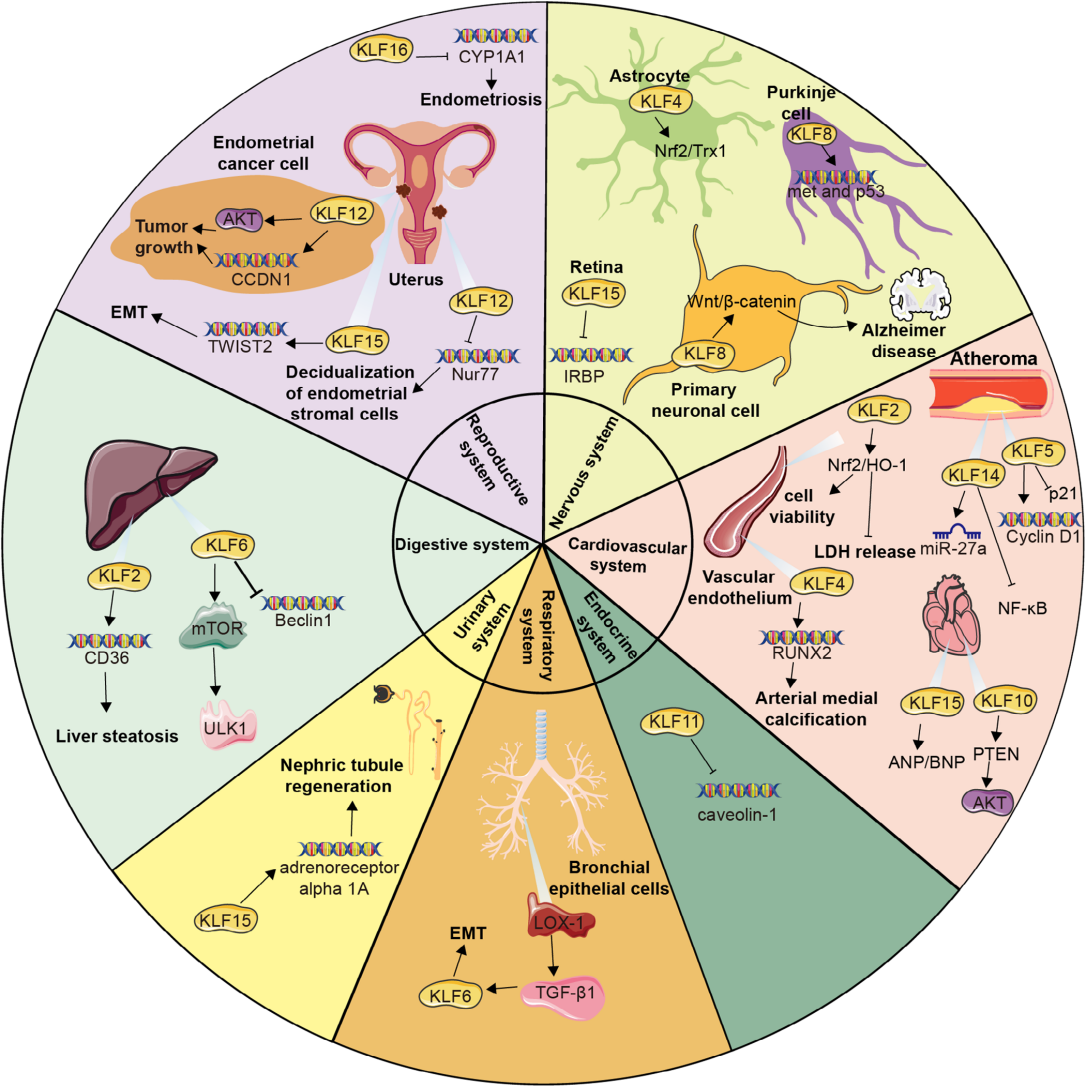
Multifaceted roles of KLFs in health and diseases across multiple systems. **Nervous system**: KLF4 activates the Nrf2/Trx1 pathway to protect neuro in astrocyte. KLF8 regulates the expression of met and p53 in purkinje cells. KLF8 also stimulates the Wnt/β‐catenin signaling pathway in the primary neuronal cells to impact the progression of Alzheimer's disease. KLF15 inhibits the transcription of the rhodopsin and IRBP in retina. **Cardiovascular system**: KLF2 enhances endothelial nitric oxide synthase uncoupling through the Nrf2/HO‐1 pathway in cases of endothelial injury to improve cell viability, decrease LDH release, and reduce the oxidative stress response. KLF4 modulates Runx2 expression to enhance the arterial medial calcification. KLF5 could activate Cyclin D1 and suppress p21 to repress apoptosis to stimulate vascular remodeling. KLF14 regulates miR‐27a to diminish lipoprotein lipase expression to inhibit atherosclerosis, as well as suppresses the NF‐κB signaling pathway to restrain endothelial inflammation. KLF10 alters Pten/Akt signaling to induce cardiomyocyte apoptosis. KLF15 regulates the expression of ANP and BNP in response to heart pressure. **Respiratory system**: LOX‐1/TGF‐β1/KLF6 signaling pathway is involved in EMT in human bronchial epithelial cells. **Digestive system**: KLF6 binds to the promoter region of Beclin1 and inhibits its transcription, as well as activates the mTOR/ULK1 pathway to protect the liver. KLF2 upregulates CD36 expression that is associated with liver steatosis. **Endocrine system**: KLF11 inhibits caveolin‐1 gene expression in response to cholesterol signals. **Urinary system**: KLF15 increases the expression of regenerative genes such as adrenoreceptor alpha 1A in Xenopus laevis to promote nephric tubule regeneration. **Reproductive system**: KLF12 activates AKT signaling and promotes CCND1 expression, contributing to tumor growth in human endometrial cancer. KLF15 promotes the transcription of TWIST2 to facilitate the process of EMT. KLF12 inhibits Nur77 expression to restrain the decidualization of human endometrial stromal cells. KLF16 suppresses the expression of endometrial CYP1A1 that is associated with endometriosis.

Furthermore, multiple studies have discovered that KLF9, KLF11, KLF12, KLF15, and KLF16 are all involved in the occurrence and development of endometriosis.^98^, ^99^ Particularly, a notable reduction in KLF15 expression was observed in the mid‐secretory epithelial endometrial cells of patients with endometriosis compared with individuals without the condition. KLF15, probably believed as a transcription factor for TWIST2, directly binds to its promoter regions to facilitate the process of EMT, orchestrating endometrial receptivity during embryo implantation.^100^ KLF11 is abundantly expressed in reproductive tissues, where it alters endometrial metabolism by colocalizing with and recruiting the corepressor SIN3A/HDAC.^101^ Research has also shown that KLF11 not only hinders the development of endometriotic lesions but also effectively suppresses pathological scarring with Collagen1 repression.^102^ KLF9 knockdown may underlie progesterone resistance in endometriosis.^103^ KLF12 restrains the decidualization of human endometrial stromal cells by inhibiting Nur77 expression.^104^ Besides, KLF16 has been found to suppress endometrial CYP1A1 expression, which is associated with endometriosis and leads to decreased enzymatic activity^105^ (Figure 2).

## 4 THE CRUCIAL ROLE OF KLFs IN BONE HOMEOSTASIS

### 4.1 The direct modulatory effects of KLFs on regulating bone homeostasis

Current studies demonstrated that KLFs could directly affect the differentiation and activity of OBs and OCs by regulating certain gene expression and signaling pathways.

Certain KLFs are identified as critical regulators in OB‐lineage cells.^106^ KLF2 promotes the expression of the osteoblastic differentiation marker genes including Alp, Osx, and Ocn, as well as stimulates mineralization through upregulating Runx2 expression at both mRNA and protein levels.^107^ Additionally, KLF10 also directly binds to regulatory elements located in the proximal region of the P1 promoter to activate Runx2 expression, as well as interact with Runx2 protein to increase the transcriptional activity.^45^ Gao et al.^108^ found that KLF5 promotes proliferation and osteogenesis differentiation of human periodontal ligament cells subjected to cyclic tensile stress through fibroblast growth factor (FGF2)‐glycogen synthase kinase 3 beta (GSK‐3β)/β‐catenin signaling pathway. Similarly, Lin et al.^109^ demonstrated that KLF4 promotes odontoblastic differentiation of dental papilla mesenchymal cells through the regulation of dentin matrix protein 1 (DMP1) and modulation of transforming growth factor‐β (TGF‐β) signaling pathways. Respectively, KLFs can also serve as downstream regulatory factors to participate in osteogenesis. It has been reported that interferon regulatory protein 2‐binding protein 2 mediates the differentiation of OCs and OBs by targeting KLF2, thus affecting bone homeostasis.^110^ Yu et al.^111^ found that conditional knockout of Cullin 4B (CUL4B) in mesenchymal stem cells (MSCs) can lead to impaired bone development along with low bone mass and reduced bone formation. Mechanistically, CUL4B suppresses KLF4 expression by directly binding to genes, epigenetically blocking the transcription, which promotes osteogenesis and inhibits adipogenesis of MSCs.^111^ Interestingly, many members of the KLF family display the opposite role in osteogenic differentiation, inhibiting bone formation and mineralization. Kim et al.^112^ found that KLF4 overexpression inhibits differentiation and mineralization of OBs. According to mechanistic studies, KLF4 acts as a negative regulator of Runx2, involved in the repression of the transcriptional process and DNA binding activity of Runx2 on target genes, including Alp and BSP.^112^ Additionally, Weng et al.^113^ demonstrated that KLF14 downregulates Wnt3A expression by binding to the BTE2 locus in the gene promoter, abolishing Wnt3A mediated Wnt/β‐catenin pathway activity and resulting in cell cycle arrest, which negatively regulates human bone marrow mesenchymal stem cells proliferation and osteogenic differentiation. Similarly, KLF15 elicited by glucocorticoid (GC) also inhibits bone formation in vivo by downregulating the Wnt signaling pathway, as well as upregulating peroxisome proliferator‐activated receptor (PPAR)γ and FoxO3a.^114^, ^115^ KLF12‐mediated inactivation of Wnt/β‐catenin signaling is also involved in the inhibition of osteogenic differentiation.^116^

As a matter of fact, KLF2 is also involved in osteoclastic differentiation, promoting bone formation and attenuating bone resorption.^117^, ^118^ Mechanistically, KLF2 inhibits osteoclastic differentiation by downregulating the expression of OC‐related genes such as c‐Fos, NFATc1, and TRAP.^110^ Laha et al.^119^ showed that KLF2 downregulates Becn1 expression by reducing the levels of histone activation marks H4K8 and H3K9 acetylation in the promoter region of Becn1, leading to the alleviation of autophagy during osteoclastic differentiation. KLF4 is also involved in the negative regulation of OC differentiation. The overexpression of KLF4 in OBs results in the downregulation of RANKL expression via directly interacting with the RANKL promoter region to prevent the vitamin D receptor (VDR) from binding to the structure, which in turn diminishes the differentiation of OCs induced by 1,25(OH)_2_D_3_ in coculture of OBs and mouse bone marrow cells.^112^ Additionally, Cicek et al.^120^ found that KLF10 inhibits OC differentiation by suppressing the activation of NFATc1 pathway as well as repressing PKB and mitogen‐activated protein kinase (MEK)/ERK signal transduction to reduce OC survival. On the contrary, Chen et al.^121^ discovered that KLF7 is capable of promoting the differentiation process by directly suppressing the expression of heme oxygenase‐1 (HO‐1), a negative regulator of OC differentiation. The phenomenon of the KLF family simultaneously regulating both osteoclastogenesis and osteoblastogenesis demonstrated a complex and balanced control required for maintaining bone homeostasis. This dual modulation reflected the multifunctional nature of KLF transcription factors, acting as pivotal nodes that integrate various signaling pathways to coordinate the actions of OCs and OBs. Such dual functionality maintained the exact modulation of bone resorption and formation processes, preventing pathological states including excessive bone destruction or formation. Dysregulation of KLF‐mediated control can disrupt this balance, leading to conditions that either promote osteolytic or osteoblastic lesions. For instance, in prostate cancer (PCa), which typically results in osteoblastic bone metastases, upregulated KLFs can enhance OB activity, leading to abnormal bone formation and sclerosis. Conversely, in breast cancer, where osteolytic metastases are more common, altered KLFs can ramp up OC activity, leading to significant bone loss and the formation of destructive lesions. Overall, the ability of KLFs to concurrently influence both cell types demonstrates their potential as therapeutic targets in bone‐related disorders.

Recent studies demonstrated that many members of KLF proteins participate in the regulation of cartilage homeostasis. KLF15 has been proven to take an active part in promoting chondrogenic differentiation of hMSCs through activating SOX‐9 expression.^122^ KLF4 regulates membranous and endochondral ossification by precisely coordinating a series of cellular functions including the differentiation and migration of chondrocytes, OBs, and vascular endothelial cells.^123^ Mechanistically, KLF4 overexpression accelerates type II collagen and SOX‐9 expression and promotes sulfated‐proteoglycan synthesis.^124^ On the contrary, KLF5 overexpression reduces the expression of type II collagen and sulfated proteoglycan at the transcriptional and translational levels, leading to the dedifferentiation of chondrocytes.^125^ Meanwhile, Shinoda et al.^126^ demonstrated that mice with heterozygous deficiency (KLF5^+/−^) exhibit impaired cartilage matrix degradation, delaying the replacement of cartilage with bone at the primary ossification center in the embryonic limbs. KLF5 induces transcriptional expression of MMP‐9, leading to degradation of the cartilage matrix and ultimately contributing to endochondral ossification.^126^ Dexamethasone, a type of GC drug, can induce apoptosis in growth plate chondrocytes and impair their differentiation, cell proliferation, and overall bone growth when used long‐term. KLF2 has been demonstrated to promote proliferation and inhibit apoptosis of Dex‐induced GPCs by targeting the Runx2‐mediated PI3K/AKT and ERK signaling pathways.^127^ All the above demonstrate that KLFs and downstream effector proteins construct a complicated transcriptional regulatory network that determines the differentiation and functions of OBs, OCs, and chondrocytes, collectively modulating skeletal development and homeostasis (Table 1).

**TABLE 1:** The modulatory effect of KLFs on bone cells in bone homeostasis.

Isotype	Target cells	Mechanism	References
KLF2	Preosteoblast MC3T3‐E1 cells	Promotes the expression of the osteoblastic differentiation marker genes Alp, Osx, and Ocn	^107^
		Increases Runx2 expression at both the mRNA and protein levels to stimulate mineralization	^107^
	Osteoclast precursor cells	Downregulates the expression of osteoclast‐related genes such as c‐Fos, NFATc1, and TRAP to inhibit osteoclast differentiation	^110^
	Chondrocytes	Directly binds to cartilage signature genes including COL2A1, COL11A2, SOX‐9, and PRG4 to enhance the expression	^128^
		Involved in PKA–RAP1–MEK–CREB signaling axis to suppress inflammation mediators and ECM‐degrading enzymes	^128^
KLF4	MSCs	Suppressed by CUL4B transcriptionally, promoting osteogenesis and inhibiting adipogenesis of MSCs	^111^
		Upregulated by SOX5. TNF‐α‐mediated upregulation of SOX5 decreases Runx2 and promotes KLF4 expression, inhibiting the osteogenic differentiation of MSCs.	^129^
	Osteoblasts	Represses the transcriptional and DNA binding activity of Runx2 on target genes such as Alp and BSP	^112^
		Directly interacts with the RANKL promoter to prevent VDR from binding to the RANKL promoter region, thereby repressing RANKL expression	^112^
	Osteoclasts	Attenuates 1,25(OH)_2_D_3_‐induced osteoclast differentiation in coculture of mouse bone marrow cells and osteoblasts through the downregulation of RANKL expression	^112^
	Chondrocytes	Directly binds to cartilage signature genes including COL2A1, COL11A2, COMP, PRG4, and SOX‐9 to enhance the expression	^128^
		Involved in PKA–RAP1–MEK–CREB signaling axis to suppress inflammation mediators and ECM‐degrading enzymes	^128^
		Regulates InsR to control JAK2/STAT3 signaling, suppressing IL‐1β‐induced apoptosis of chondrocytes	^130^
	Dental papilla mesenchymal cells	Regulates DMP1 and modulates TGF‐β signaling pathways to promote dentinogenesis	^109^
KLF5	Chondrocytes	Reduces the expression of type II collagen and sulfated proteoglycan at the transcriptional and translational levels, leading to the dedifferentiation of chondrocytes	^125^
		Induces MMP‐9 expression at transcriptional level to cause cartilage matrix degradation during endochondral ossification	^126^
	Periodontal ligament cells	Regulates FGF2–GSK‐3β/β‐catenin signaling pathway to promote proliferation and osteogenesis differentiation of human periodontal ligament cells subjected to cyclic tensile stress	^108^
KLF7	Osteoclasts	Inhibits HO‐1 to enhance osteoclast differentiation	^121^
KLF10	Osteoblasts	Binds to the P1 promoter to activate Runx2 expression	^45^
	Osteoclast precursor cells	Reduces the activation of NFATc1 pathway to control osteoclast differentiation	^120^
	Osteoclasts	Suppresses PKB and MEK/ERK signaling to reduce osteoclast survival	^120^
	Chondrocytes	Inhibition of KLF10 ameliorates ROS production and maintains mitochondrial homeostasis to protect chondrocytes	^131^
KLF11	Chondrocytes	Inhibits p38 MAPK signaling pathway to suppress oxidative stress and apoptosis	^132^
KLF12	Human umbilical cord blood‐derived MSCs	Mediates inactivation of Wnt/β‐catenin signaling to repress osteogenic differentiation	^116^
KLF14	MSCs	Downregulates WNT3A expression by binding to the BTE2 locus to abolish Wnt/β‐catenin pathway activity	^113^
KLF15	Osteoblasts	Downregulates WNT3A and upregulates PPARγ and FoxO3a under the elicitation of GC to inhibit bone formation	^114^
	MSCs	Activates SOX‐9 to promote chondrogenic differentiation of hMSCs	^122^
	Chondrocytes	Downregulates MMP‐3 expression to inhibit the degradation of type II collagen and aggrecan	^133^

### 4.2 The indirect effects of KLFs on bone homeostasis via energy metabolism

Over the last two decades, numerous studies have emphasized the crucial significance of KLFs in metabolic processes, which ultimately affect cellular physiological activities and the occurrence and development of diseases.^134^, ^135^ It is well known that bone is a highly metabolized tissue and needs to undergo metabolic reprogramming during bone remodeling to satisfy the high energy demand involved in many aspects including cell migration, cytoskeleton remodeling, differentiation, and secretion. ^136^ Therefore, it can be inferred that KLF proteins potentially play significant roles in the regulation of bone remodeling by fine‐tuning local metabolic programs to precisely match energetic demand, thereby modulating the dynamic balance of the bone matrix.

#### 4.2.1 KLFs mediate bone homeostasis by regulating glycometabolism

Glycometabolism is widely recognized for its function as a primary energy source, encompassing three main metabolic pathways: oxidative phosphorylation (OXPHOS), glycolysis, and the pentose phosphate pathway.^137^ To be specific, glucose is predominantly broken down through glycolysis and OXPHOS to produce ATP for energy provision. The pentose phosphate pathway supports cell growth by delivering the fundamental materials needed for macromolecule synthesis.^138^ Accumulating evidence suggested that glycometabolism is indispensable for the activity and function of OBs and OCs. OBs are highly metabolically active cells that require substantial amounts of energy to synthesize the organic matrix of bone. Glycolysis is the main energy source of immature OBs, which stimulates the anabolic response of bone to parathyroid hormone and Wnt proteins including Wnt7B and Wnt3A in OB lineage cells.^139^, ^140^, ^141^, ^142^ Additionally, glucose transporter (GLUT) 1, GLUT3, and GLUT4 have been found to express in OBs.^143^, ^144^, ^145^ GLUT1 is the primary glucose transporter in primary OBs, which promotes OB differentiation and bone formation by suppressing AMPK‐dependent Runx2 degradation.^146^ OCs, as the multinucleated cells responsible for bone resorption, are also dependent on glycolysis and OXPHOS for their energy demands. As OC differentiates, the number of mitochondria increases, accompanied by a higher OCR and elevated expression of glycolysis‐related enzymes, such as hexokinase, phosphofructokinase, and pyruvate kinase. Additionally, the enzymes involved in the TCA cycle and OXPHOS are upregulated, indicating a surge in energy production.^147^ Collectively, glucose metabolism regulates bone homeostasis by providing essential energy and intermediates for OB and OC function, thus influencing bone formation and resorption balance.

Recent studies demonstrated that KLF15 overexpression in adipocytes and myocytes promotes transcription of the insulin‐sensitive GLUT, thereby facilitating the uptake of glucose into cells.^148^ Furthermore, Yang et al.^149^ demonstrated that KLF14 facilitates the phosphorylation of insulin receptor (InsR), which enhances insulin‐mediated glucose uptake. The uptake of glucose can stimulate cells to produce ATP, which plays a crucial role in OB proliferation through increasing the expression of Alp, Runx2, and Osteocalcin during the initial phases of maturation in vitro.^150^ This process is facilitated by elevated levels of intracellular Ca^2+^ signals, which activate the NFAT transcription factor during osteogenic differentiation. ATP enhances osteogenesis as indicated by Ca^2+^ deposition, which was consistent with the upregulation of OB genes such as BMP2, MMP‐13, Col3a1, Ctsk, Flt1, and Bgn.^151^ Additionally, the vesicular release of ATP from human bone marrow stromal cells increases intracellular calcium concentration, in turn activating calcineurin and promoting the nuclear translocation of NFATc1, which directly orchestrates behavior of OBs and osteocytes.^152^ The ATP‐dependent Ca^2+^ signals have the potential to enhance osteogenic differentiation protocols in tissue‐engineered bone.

Studies have shown that in response to reactive oxygen species (ROS), cAMP response element–binding protein (CREB) induces the transcription of Ppargc1b that encodes for PPARγ coactivator 1 (PGC‐1) during OC differentiation. Knockdown of Ppargc1 in vitro inhibits OC differentiation and mitochondria biogenesis, whereas deletion of the Ppargc1b gene leads to increased bone mass due to impaired OC function. Further studies have also observeddefects in PGC‐1‐deficient OBs, consistent with the aforementioned experimental findings both in vivo and in vitro. During OC development, the demand for iron is significant and is primarily met through the transferrin receptor 1 (TfR1), which facilitates OC differentiation by inducing mitochondrial respiration, increasing the production of ROS, and accelerating Ppargc1b transcription.^153^ Myc has been evidenced to facilitate osteoclastogenesis via driving metabolic reprogramming during OC differentiation and functioning as a metabolic switch to an oxidative state, as well as being involved in the transcriptional induction of ERRα, a nuclear receptor that cooperates with the transcription factor NFATc1.^154^ The Volker Ellenrieder group proved that KLF11 participates in TGF‐β‐mediated c‐Myc gene expression in cancer, which proposes new ideas for bone tumors.^155^

**FIGURE 3:**
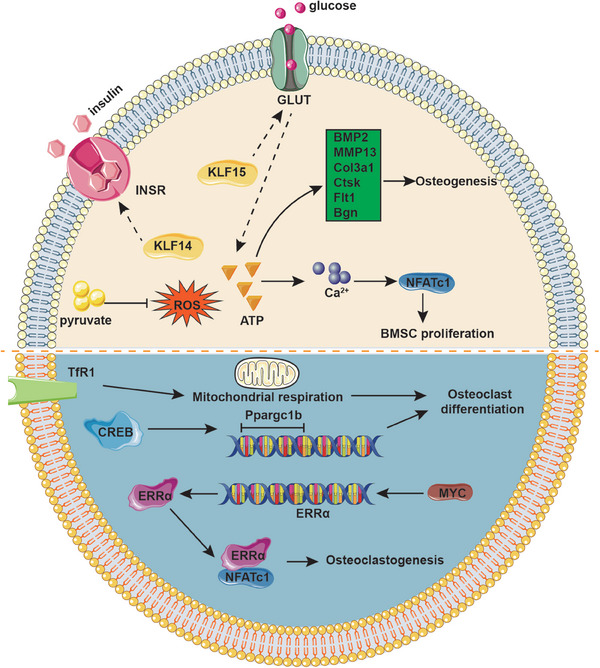
Glycometabolism model involving KLFs in bone homeostasis. Pyruvate produced in glycometabolism could suppress hydrogen peroxide‐induced generation of intracellular ROS in OBs. KLF15 and KLF14 may promote GLUT and InsR, facilitating glucose uptake to supply more ATPs. ATPs produced by oxidative phosphorylation upregulate OB genes such as BMP2, MMP13, Col3a1, Ctsk, Flt1, and Bgn to enhance osteogenesis and increase intracellular calcium concentration, in turn promoting the nuclear translocation of NFATc1 to enhance BMMSC proliferation. CREB induces the transcription of Ppargc1b in response to ROS during OC differentiation. Moreover, Myc induces the transcription of ERRα, which cooperates with NFATc1 to facilitate osteoclastogenesis. Increased iron is transported by TfR1, inducing mitochondrial respiration and facilitating OC differentiation.

Additionally, the reprogramming of gluconeogenesis and anaerobic glucose metabolism mediated by KLFs alters the content of the energy molecule pyruvate. Previous studies have shown that pyruvate plays a crucial role in maintaining cell viability and survival of proliferative cultured calvarial OBs.^156^ As an important intermediate product of glycometabolism, pyruvate could suppress hydrogen peroxide‐induced generation of intracellular ROS in OBs, performing cytoprotective roles via a mechanism associated with the antioxidative property.^157^ Additionally, Singh et al.^158^ identified that stromal precursor cells deficient in Sod2 or intrinsically aged experience toxic accumulation of alpha‐ketoglutarate due to impairment in pyruvate metabolism, which suppresses both osteogenic and adipogenic differentiation. With the deepening of research, the indirect regulatory pathway of KLFs affecting pyruvate content by altering anaerobic metabolism has gradually become clear in bone homeostasis, but its core mechanism still needs further confirmation (Figure 3).

#### 4.2.2 KLFs mediate bone homeostasis by regulating lipid metabolism

The majority of lipids in bone are present within the bone marrow, not only serving as energy storage tissue but also transducing multiple signals. The key role of lipid metabolism in cell function and differentiation of the skeletal system has long been appreciated. Clinical studies have demonstrated that patients with osteoporosis (OP) often exhibit lipid metabolism dysfunction to some extent, and simultaneously obese patients display heightened OC activity, increased OC biomarker activity, and elevated levels of bone destruction products.^159^ There is a well‐established connection between excessive lipid accumulation and bone loss, potentially linked to the activation of OCs and the inhibition of OBs.^160^ As the essential factors to maintain bone homeostasis, long‐chain polyunsaturated fatty acids (PUFAs) and their metabolites widely participate in the differentiation and metabolism of bone cells and chondrocytes. 15‐deoxyΔ12,14‐prostaglandin J2, an endogenous prostaglandin, has been proven to promote adipogenesis by binding to PPARγ and inhibiting osteoblastogenesis by blocking the Wnt/β‐catenin signaling pathway.^161^ The arachidonic acid and docosahexaenoic acid prominently restrain RANKL‐induced OC differentiation in a dose‐dependent manner.^162^ The palmitic acid (PA) also enhances OC differentiation and the supplementation with oleic acid could prevent PA‐induced osteoclastogenesis, playing a protective role for bone health.^163^ Additionally, omega‐3 PUFAs especially α‐linolenic acid decrease the expression of MMP‐13 and ADAMT5 and suppress oxidative stress and chondrocyte apoptosis via NF‐κB and inducible nitric oxide synthase pathways.^164^ Furthermore, various studies have indicated that KLFs play diverse and essential roles in the management of lipid metabolism. Uncontrollable KLF expression owing to senescence, stress, and diseases may result in a disorder of fatty acid metabolism, thus affecting the metabolic adaptation of bone cells and destroying bone homeostasis. Mitochondrial FAO is responsible for energy provision during fasting, febrile illness, and strenuous activities, and approximately 80% of energy for the heart and liver is supplied by FAO.^165^ KLF4 and KLF5 could enhance the activity of the PPARa promoter region and promote its expression, thereby contributing to lipid oxidation and FAO.^166^, ^167^, ^168^ As another significant member of the PPAR family, PPARδ increases lipid utilization by enhancing mitochondrial function and fatty acid desaturation pathways, serving to mediate muscle fiber type and endurance exercise adaptation.^169^ Oishi et al.^170^ demonstrated that KLF5 participates in the formation of transcriptional repression complexes with PPAR and corepressors, which inhibits the expression of genes involved in lipid oxidation and energy uncoupling, such as carnitine‐palmitoyl transferase‐1b (Cpt1b) and uncoupling proteins 2 and 3 (Ucp2 and Ucp3). Additionally, KLF15 colocalizes with PPARδ on the skeletal muscle genome, and promotes the recruitment of PPARδ to the correct transcriptional loci, regulating the transcriptional expression of a range of downstream genes relevant to lipid metabolism in an interdependent manner.^171^ KLFs serve as critical contributors to regulating lipid metabolism, further impacting the whole motor system. Taken together, KLFs have the potential to regulate bone homeostasis through lipid metabolism, primarily involving the accumulation of lipids, as well as the synthesis and oxidation of fatty acids.

#### 4.2.3 KLFs mediate bone homeostasis by regulating amino acid metabolism

Amino acids are indispensable for diverse cellular functions and serve as crucial sources of energy. The synthesis and breakdown of these molecules play pivotal roles in maintaining bone homeostasis.^172^, ^173^ Numerous studies have explored the regulatory role of KLFs in amino acid metabolism. Generally, amino acids derived from muscle proteins are catabolic to pyruvate by Pepck and pyruvate kinase during starvation, and subsequently transported to the liver for gluconeogenesis.^174^ KLF15, highly expressed in metabolically dynamic tissues such as the liver, heart, and skeletal muscle, simultaneously regulates the expression of genes related to gluconeogenic or amino acid‐degrading enzymes.^148^, ^175^ Gray et al.^176^ demonstrated that KLF15 depletion leads to defective amino acid catabolism in skeletal muscle, which limits the availability of gluconeogenic substrate and ultimately causes severe hypoglycemia in mice. Collectively, KLF15 utilizes the amino acid metabolism of skeletal muscle to promote glucose production in the liver, thereby maintaining the energy supply during starvation.^176^ Amino acids are converted into fatty acids, glucose, and other molecules for efficient energy production through various biochemical reactions. The energy supply of amino acids is equally important for bone cell growth and metabolism. Previous studies have demonstrated that the amine group on branched‐chain amino acids (BCAAs) can be transferred by branched chain amino transfers 1 (BCAT1) to alpha‐ketoglutarate, which produces glutamate and ultimately be oxidized to provide energy for cells α‐ketonic acid. Go et al.^177^ have discovered the level of BCAA increased steadily in the process of OC, and selective inhibition of BCAT1 with gabapentin could significantly reduce the maturation of OC, which indicates that BCAA metabolism involves the regulation of OC differentiation. Furthermore, amino acids can also act as precursors of bioactive molecules in bone formation and resorption. For instance, arginine enhances osteogenesis in hMSCs through upregulation of osteogenic transcription factors such as Runx2, DLX5, and Osterix.^178^ As the precursor of l‐cysteine and reduced glutathione, N‐acetyl‐l‐cysteine (NAC) exhibits high antioxidant capabilities that inhibits cell apoptosis and maintain cell survival.^179^ Recent studies demonstrated that NAC promotes the proliferation and osteogenic differentiation of rat femur bone marrow‐derived OB‐like cells through suppression of ROS, which accelerates bone regeneration in rats with severe bone defects.^180^, ^181^ Additionally, pretreatment with NAC suppresses RANKL‐induced OC differentiation in bone marrow‐derived monocytes.^182^ Karner et al.^183^ found that Wnt activation promotes glutamine catabolism, which enhances the expression of genes involved in amino acid supply, transfer RNA aminoacylation, and protein folding, thereby meeting the requirement of increased energetic and synthetic needs related to mature OBs. These findings indicate that KLFs may participate in the regulation of amino acid metabolism, which is essential for bone development and homeostasis. However, further researches are required to confirm this hypothesis.

## 5 THE COUPLING EFFECTS AMONG KLFs AND EPIGENETIC REGULATIONS DURING BONE REMODELING

Increasing evidence demonstrated that epigenetic mechanisms, involved in regulating chromatin state and transcript metabolism, work in conjunction with KLFs to regulate bone remodeling, which results in the formation of a complex gene regulatory network.^184^ Here, we highlighted recent progress in understanding the epigenetic pathways that regulate the expression and function of KLFs during bone remodeling, and additionally discussed the reciprocal regulation of KLFs on epigenetic regulators, including DNA modification, histone modification, and noncoding RNA (ncRNA).

### 5.1 DNA modification

DNA modification involves chemically altering DNA molecules to regulate gene expression and cellular function, which includes modifications like methylation, hydroxymethylation, and phosphorylation. During bone homeostasis, DNA modification impacts bone formation and absorption by controlling gene expression and signaling pathways in bone cells. Specifically, DNA methylation is intimately associated with cell differentiation and function regulation.^185^ DNA methylation, a chemical modification process in which S‐adenosylmethionine serving as a methyl donor covalently binds to specific bases on a DNA sequence under the catalysis of DNA methyltransferase (DNMT), is regarded as one of the most representative epigenetic modifications closely related to human development.^186^ Previous studies have demonstrated that the alterations in promoter can impact the activity of OB and OC, disequilibrating osteogenesis and osteoclastogenesis.^187^ It has been established that the methylation status contributes to determining cell lineage commitment. Typically, the methylation levels in promoter regions exhibit an inverse correlation with gene expression. Specifically, the promoter regions of osteogenic‐related genes like Runx2 and osteocalcin are found to be completely hypomethylated in BMMSCs,^188^ while the promoters of other osteogenic lineage‐specific genes, such as ROR2, Dlx5, Runx2, and osterix, become demethylated during osteogenic differentiation, leading to increased gene expression in mRNA level.^189^ Apart from its known function in osteogenic lineage differentiation, DNA methylation also plays a significant role in osteoclastogenesis. Studies have shown that OC formation is reduced in bone marrow macrophage precursor cells deficient in DNMT3a. Moreover, OC‐specific DNMT3a knockout mice display high bone mass phenotypes and fewer osteoporotic possibilities compared with wild‐type mice.^190^

Recently, significant advances have been made in the effect of DNA methylation on KLF expression. It has been demonstrated that DNMT3B upregulation plays a role in mediating KLF5 hypermethylation induced by oxidative stress, which ultimately blocks osteogenic differentiation by reducing the expression and nuclear translocation of β‐catenin.^191^ Yu et al.^111^ demonstrated that CUL4B–RING E3 ligase (CRL4B) epigenetically represses KLF4 transcription such as DNA methylation and histone methylation through coordination with enhancer of zeste homolog 2 (EZH2), contributing to bone formation.

### 5.2 Histone modification

#### 5.2.1 Histone methylation

Histone modifications include methylation, acetylation, phosphorylation, and so on, among which histone methylation is the most common modification in eukaryotic cells.^192^, ^193^ Substantial research has proved that during the differentiation and maturation of OBs and OCs, several genes are modulated by HMTs or histone demethylases, which take central parts in the regulation of histone methylation.^194^, ^195^ For instance, Suv39h1, as a methyltransferase, catalyzes the dimethylation or trimethylation of H3K9, leading to its binding to the promoter of Runx2, which inhibits the transcription of Runx2, consequently delaying OB differentiation.^196^ On the other hand, EZH2 functions as a trimethyltransferase of H3K27 facilitating the formation of H3K27me3 to activate the transcription of the Wnt4 gene in OBs, ultimately promoting osteogenic differentiation.^197^ Based on previous research, the expression of crucial genes involved in bone homeostasis can be affected by alterations in the level of histone methylation, which has been speculated to be associated with the action of KLFs. It is confirmed that the KLF2 expression is upregulated during osteogenic differentiation, which activates the ATG7 gene via collaboration with H3K27Ac and H3K4me3 in the promoter region of ATG7.^198^ The extensive trimethylation of histone H3 lysine 4 (H3K4me3) as well as histone H3 lysine 27 (H3K27me3) occupy the promoters of KLF4, which is closely associated with the monocyte‐into‐phagocyte differentiation program.^199^ Additionally, recent research has demonstrated the role of chromatin in the control of DNA methylation. Inactivated histone methylation, especially trimethylation of H3 at K9, has been proven to promote DNA methylation, which suggests the involvement of chromatin remodeling factors in DNA methylation.^200^ Further studies are required to determine whether similar regulatory mechanisms occur in OC differentiation.

#### 5.2.2 Histone acetylation

Maity et al.^198^ found that KLF2 binds to the promoter region of the autophagic molecule ATG7 through the upregulation of active epigenetic marks H3K27Ac and H3K4me3, thereby inducing mitophagy and altering mitochondrial metabolism during osteoblastic differentiation. Besides, Das et al.^201^ demonstrated that KLF2 depletion promotes the enrichment of active histone marks H3K9Ac and H4K8Ac and HATs (P300, PCAF) on the MMP‐9 promoter region in monocytes. As mentioned above, KLF4 promotes odontoblastic differentiation by increasing DMP1 expression. A recent study further proved that HDAC3 and P300 are enriched on the promoter region of KLF4 target genes DMP1 and Sp7, and interact with KLF4 in a temporal‐specific manner, ultimately modulating DMP1 and Sp7 transcription and promoting dentinogenesis and odontoblastic differentiation.^202^ Cancer stem cells (CSCs) play essential roles in tumorigenesis, recurrence, and therapy resistance. The Yicun Wang group identified that KLF11 serves as a negative regulator in sarcoma CSCs and histone acetylation may participate in the detailed modulation process. Mechanistically, KLF11 restrains the stemness of osteosarcoma (OS) by recruiting SIN3A/HDAC to suppress the transcriptional output of yes‐associated protein (YAP)/transcriptional enhanced associate domain (TEAD).^203^

### 5.3 Noncoding RNA

#### 5.3.1 MicroRNAs

MicroRNAs (miRNAs) are a family of small ncRNA molecules regulating gene expression by targeting the mRNA at the posttranscriptional level through either mRNA cleavage or translational inhibition via direct binding the 3′ untranslated region (3′‐UTR), thereby controlling cell proliferation, differentiation, and apoptosis.^204^, ^205^, ^206^ Recent studies demonstrated that miRNAs take an active part in the maintenance of bone metabolic balance. Mineralizing OB‐derived exosomes serve as carriers of miRNAs related to OB differentiation and mediate activation of Wnt signaling via Axin1 inhibition, thereby promoting MSC osteogenic differentiation.^207^ Besides, miRNA‐mediated intercellular communication also plays a significant role in the intricate interaction between bone cells and muscle cells. Qin et al.^208^ demonstrated that myostatin, a myokine secreted by muscles, suppresses miR‐218 expression, which in turn releases the inhibition of sclerostin in osteocytes. The decreased osteocytic exosomes containing miR‐218 are found to downregulate osteoblastic differentiation via inactivation of the Wnt signaling pathway and reduction of Runx2.^208^ MiRNAs also play a role in regulating bone homeostasis by targeting KLFs.

KLF10 has been proven to promote OB differentiation, bone formation, and mineralization. You et al.^209^ found that miR‐197‐3p significantly represses KLF10 expression, thereby inhibiting OB differentiation and disrupting the metabolic balance of bone. Furthermore, miR‐20a‐5p was demonstrated to promote adipocyte differentiation from bone marrow stromal cells by negatively regulating KLF3 during the early phase of adipogenesis.^210^ MiRNA‐1236‐3p expression has been found to decrease in OS, which inhibits the proliferative ability of OS cells and mediates apoptosis by targeting KLF8.^211^ In conclusion, a program of multiple miRNAs may control cellular lineage progression and function by affecting KLFs, thereby directly or indirectly modulating bone homeostasis.

#### 5.3.2 Long ncRNAs

Recent research evidenced that long ncRNAs (lncRNAs) function as key regulators by modulating the expression level of target genes via epigenomic, transcriptional, or posttranscriptional approaches in crucial cellular functions, including cell proliferation, differentiation, apoptosis, migration, and invasion.^212^, ^213^ Yang et al.^214^ identified a novel mutation in the lncRNA Reg1cp, which promotes bone formation and leads to higher bone density. Mechanistically, mutated lncRNA Reg1cp directly binds to KLF3 to inhibit its activity, thereby promoting the formation of CD31^hi^Emcn^hi^ endothelium in the bone marrow and thus stimulating angiogenesis during osteogenesis.^214^ Additionally, Recent studies have shown that lncRNAs play a role in regulating the expression of KLFs by acting as sponges for specific miRNAs. For example, small nucleolar RNA host gene 15 (SNHG15) has been found to sponge miR‐7, which targets KLF4, leading to the inhibition of ECM degradation and ultimately reducing the progression of osteoarthritis (OA).^215^ LncRNA maternally expressed 3 (MEG3) was proved to improve KLF4 expression via sponging miR‐9‐5p, which significantly enhances the survival and migration ability of chondrocytes, and inhibits cell apoptosis and inflammatory response, thereby protecting cartilage.^216^ LncRNA LINC02381 suppresses osteogenic differentiation of human umbilical cord blood‐derived MSCs by sponging miR‐21 to enhance the inactivation of the Wnt/β‐catenin pathway mediated by KLF12.^116^ Furthermore, lnc‐HLA‐DQA1‐5, lnc‐RP11‐127H5.1.1‐1, and lnc‐RTN2‐1 regulate KLF2 expression by sponging miRNAs (miR‐6799‐5p, miR‐1915‐3p, miR‐6764‐5p, miR‐6796‐5p, and miR‐6895‐3p), which is involved in the occurrence and development of meniscus degeneration in OA.^217^

#### 5.3.3 Circular RNAs

**FIGURE 4:**
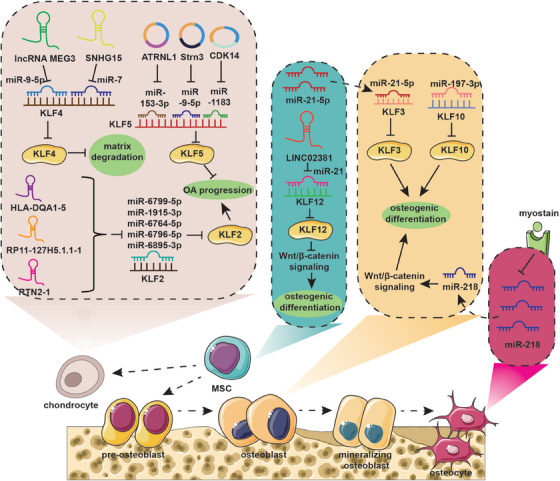
The coupling effects between KLFs and ncRNAs during bone remodeling. MiR‐21‐5p derived from BMMSCs inhibits KLF3 to enhance OB proliferation. Osteocyte‐derived exosomal miR‐218, which activates the Wnt signaling pathway and induces Runx2, is suppressed in osteocytes due to myokine secreted by muscles, resulting in the downregulation of osteoblastic differentiation. MiR‐197‐3p represses KLF10 expression, thereby inhibiting OB differentiation and disrupting the metabolic balance of bone. LncRNA LINC02381 suppresses osteogenic differentiation of human umbilical cord blood‐derived MSCs by sponging miR‐21 to enhance the inactivation of the Wnt/β‐catenin pathway mediated by KLF12. SNHG15 sponges miR‐7 targeting KLF4 and lncRNA MEG3 sponges miR‐9‐5p targeting KLF4, thereby inhibiting ECM degradation and cell apoptosis. lnc‐HLA‐DQA1‐5, lnc‐RP11‐127H5.1.1‐1, and lnc‐RTN2‐1 regulate KLF2 expression by sponging miRNAs (miR‐6799‐5p, miR‐1915‐3p, miR‐6764‐5p, miR‐6796‐5p, and miR‐6895‐3p), which is involved in the occurrence and development of meniscus degeneration. Circ‐Strn3 could sponge miR‐9‐5p targeting KLF5 and Circ‐ATRNL1 could sponge miR‐153‐3p targeting KLF5 and circ‐CDK14 could sponge miR‐1183 targeting KLF5, thereby ameliorating inflammatory responses, cell apoptosis, and ECM degradation.

Circular RNAs (circRNAs), derived from the process of back‐splicing, are more stable than linear RNAs because of their unique loop structure, which is resistant to exonuclease‐mediated degradation.^218^, ^219^ It has been demonstrated that circRNAs contain abundant miRNA binding sites and could function as competing endogenous RNAs to modulate the interaction between miRNA and target mRNA.^220^, ^221^, ^222^ KLFs serve as downstream protein of miRNAs and their expression depends on the precise regulation of circRNA/miRNA interaction network. As mentioned above, KLF5 promotes cartilage matrix degradation by inducing MMP‐9 expression at the transcriptional level.^126^ Interestingly, the Kai Fu Wang group demonstrated that circ‐ATRNL1 could directly target miR‐153‐3p to inhibit miR‐153‐3p‐mediated KLF5 silencing, thereby ameliorating inflammatory responses, cell apoptosis, and ECM degradation.^223^ Similarly, circ‐Strn3 could inhibit the matrix metabolism of chondrocytes in OA by competitively sponging miRNA‐9‐5p that targets KLF5^224^ (Figure 4 and Table 2).

**TABLE 2:** The coupling effect between KLFs and epigenetic regulation during bone remodeling.

Level	Modification	Key enzyme	Participants	Mechanism	References
Pretranscriptional modification	DNA methylation	DNMT3B	KLF5 gene	DNMT3B mediates KLF5 hypermethylation induced by oxidative stress, decreasing expression, and nuclear translocation of β‐catenin to block osteogenic differentiation.	^191^
		EZH2	CRL4B/KLF4 gene	CRL4B epigenetically represses KLF4 transcription through DNA methylation coordination with EZH2.	^111^
	Histone methylation	Unclear	KLF2/ATG7 (H3K4)	KLF2 activates the ATG7 gene through the enrichment of methylation of H3K4.	^198^
	Histone acetylation	Unclear	KLF2/ATG7 (H3K27)	KLF2 activates the ATG7 gene through the enrichment of acetylation of H3K27.	^198^
		HAT (P300, PCAF)	KLF2/MMP‐9 (H3K9 and H4K8)	KLF2 decreases the enrichment of active histone marks H3K9Ac and H4K8Ac and HAT (P300, PCAF) on the MMP‐9 promoter region along with lower migration of activated monocytes to the rheumatoid arthritis sites.	^201^
		Unclear	KLF2/Becn1 (H4K8 and H3K9)	KLF2 decreases the level of histone activation marks H4K8 and H3K9 acetylation in the promoter region of Becn1 to inhibit the expression, thereby alleviating autophagy during osteoclastic differentiation.	^119^
		HDAC3 and P300	KLF4/DMP1 and Sp7	HDAC3 and P300 are recruited on the promoter region of KLF4 target genes DMP1 and Sp7, modulating the transcription to promote dentinogenesis and odontoblastic differentiation.	^202^
		HDAC	KLF11	KLF11 substantially suppresses CSC stemness of OS by restraining YAP/TEAD transcription activity via recruiting SIN3A/HDAC.	^203^
Posttranscriptional modification	MicroRNA		MiRNA‐197‐3p/KLF10	MiRNA‐197‐3p represses KLF10 expression to inhibit osteoblast differentiation.	^209^
			MiRNA‐21‐5p/KLF3	BMSCs‐derived exosomal miRNA‐21‐5p inhibits KLF3 to enhance the proliferation and osteoblast differentiation.	^225^
			MiRNA‐20‐5p/KLF3	MiR‐20a‐5p promotes adipogenic differentiation through targeting KLF3.	^210^
			MiRNA‐135a/KLF4	MiRNA‐135a binds to 3′‐UTR of KLF4 to inhibit its translation in OS cells.	^226^
			MiRNA‐652/KLF9	MiRNA‐652 binds to 3′‐UTR of KLF9 to inhibit its translation, performing a carcinogenic role in OS cells.	^227^
			MiRNA‐378/KLF9	MiR‐378 downregulates the expression of KLF9, promoting the proliferation of OS cells.	^228^
			MiRNA‐1236‐3p/KLF8	MiRNA‐1236‐3p downregulates KLF8 to suppress the proliferative ability and induce apoptosis of OS cells.	^211^
			KLF5/miRNA‑487a/NKX3‑1	KLF5 directly binds to the promoter region of miR‐487a to enhance its expression, which targets NKX3‐1 to significantly facilitate the invasion and metastasis of OS cells.	^229^
			KLF8/miRNA‑429/SOX2	KLF8 directly binds the promoter region of miR‐429 to inhibit its expression, which targets SOX2 to mediate cancer stem cell‐like features in CD133+ OSCs.	^230^
	Long noncoding RNA		LncRNA Reg1cp/KLF3	Mutated lncRNA Reg1cp directly binds to KLF3 to inhibit its activity to promote angiogenesis during osteogenesis.	^214^
			LncRNA SNHG15/miRNA‐7/KLF4	LncRNA SNHG15 sponge miRNA‐7 to improve KLF4 expression to inhibit ECM degradation and alleviate OA progress.	^215^
			LncRNA MEG3/miRNA‐9‐5p/KLF4	LncRNA MEG3 sponge miRNA‐9‐5p to improve KLF4 expression, protecting chondrocytes.	^216^
			LncRNA LINC02381/miRNA‐21/KLF12	LncRNA LINC02381 repressed osteogenic differentiation of human umbilical cord blood‐derived MSCs through sponging miRNA‐21 to enhance the inactivation of Wnt/β‐catenin pathway mediated by KLF12.	^116^
			lnc‐HLA‐DQA1‐5, lnc‐RP11‐127H5.1.1‐1 and lnc‐RTN2‐1/miRNA‐6799‐5p, miRNA‐1915‐3p, miRNA‐6764‐5p, miRNA‐6796‐5p and miRNA‐6895‐3p/KLF2	lnc‐HLA‐DQA1‐5, lnc‐RP11‐127H5.1.1‐1 and lnc‐RTN2‐1 sponge miRNA‐6799‐5p, miRNA‐1915‐3p, miRNA‐6764‐5p, miRNA‐6796‐5p, and miRNA‐6895‐3p to regulate KLF2 in the mechanism of meniscus degeneration.	^217^
			LncRNA SNHG6/KLF2	LncRNA SNHG6 downregulates KLF2, accelerating OS progression.	^231^
			LncRNA KCNQ1OT1/miR‐3666/KLF7	KCNQ1OT1 directly targets the miR‐3666/ KLF7 axis and activates Wnt/β catenin signaling to facilitate OS progression.	^232^
	Circular RNA		CircRNA‐LRP6/KLF2	CircRNA‐LRP6 interacts with LSD1 and EZH2 to mediate their binding to the promoter regions of KLF2, inhibiting its expression and promoting OS development.	^233^
			CircATRNL1/miRNA‐153‐3p/KLF5	CircATRNL1 targets miRNA‐153‐3p to regulate KLF5 expression to protect against OA.	^223^
			CircCDK14/miRNA‐1183/KLF5	CircCDK14 enhances KLF5 expression via targeting miR‐1183, ameliorating interleukin‐1β‐induced chondrocyte damage in OA.	^234^
			CircStrn3/miRNA‐9‐5p/KLF5	CircStrn3 sponge miRNA‐9‐5p that targets KLF5 to inhibit matrix metabolism of chondrocytes.	^224^
			CircRNA_0078767/miRNA‐889/KLF9	CircRNA_0078767 upregulates KLF9 expression by targeting miRNA‐889, thereby inhibiting the progression of OS.	^235^

## 6 KLFs PARTICIPATE IN THE PROGRESSION AND DEVELOPMENT OF BONE DESTRUCTION DISEASES

### 6.1 Osteoarthritis

OA is a prevalent joint disorder that affects people worldwide caused by the excessive production of cartilage MMPs, inflammatory response, and apoptosis, leading to the destruction of the cartilage matrix, inhibition of cartilage matrix synthesis,^236^ chronic pain, and disability.^237^, ^238^ Recent research demonstrated that multiple members of the KLF family are intimately involved in the onset or progression of OA.

Kawata et al.^128^ identified that KLF4 and KLF2 are important modulators of chondrocytes. Adenoviruses containing KLF4 and KLF2 genes could significantly alleviate OA progression, including cartilage degradation, meniscus, and synovium inflammation. On the one hand, KLF4 and KLF2 are central transcription factors that directly bind to cartilage signature genes such as COL2A1, PRG4, and SOX to increase their expression. On the other hand, they are involved in protein kinase A (PKA)–RAP1–MEK–CREB signaling axis, ultimately suppressing mediators of inflammation and ECM‐degrading enzymes.^128^ Besides, KLF4 transcriptionally regulates InsR, which functions as a critical regulatory factor inactivating JAK2/STAT3 signaling, thus suppressing apoptosis of IL‐1β‐induced OA chondrocytes.^130^ Another study also demonstrated that KLF4 promotes chondrocyte differentiation induced by simvastatin, which may be involved in the protective role of KLF4 in OA cartilage.^124^ lncRNA MEG3 induces KLF4 expression by sponging miR‐9‐5p, thereby enhancing the protective role of KLF4 in chondrocytes.^216^ Similarly, SNHG15 regulates the miR‐7/KLF4/β‐catenin axis, modulating ECM homeostasis to alleviate the progression of OA.^215^ KLF2 has also been proven to block apoptosis of chondrocytes and matrix degradation by activating Nrf2/antioxidant‐response element (ARE) signaling pathway.^239^

Sun et al.^240^ showed that early growth response 1 (EGR1), which is highly expressed in OA cartilage, activates KLF5 and β‐catenin signaling to promote cartilage degeneration and hypertrophy. Interestingly, another study found that KLF5 is downregulated in OA cartilage tissues and protects chondrocytes against IL‐1β‐induced damage cell damage. The molecular mechanism suggested that circ‐CDK14 could enhance KLF5 expression via targeting miR‐1183, which serves as a negative upstream regulator of KLF5.^234^ Moreover, circStrn3 also inhibits matrix metabolism of chondrocytes in OA through competitively sponging miRNA‐9‐5p targeting KLF5.^224^ Circ‐ATRNL1/miR‐153‐3p/KLF5 axis also plays a protective role in the development and progression of OA.^223^

**FIGURE 5:**
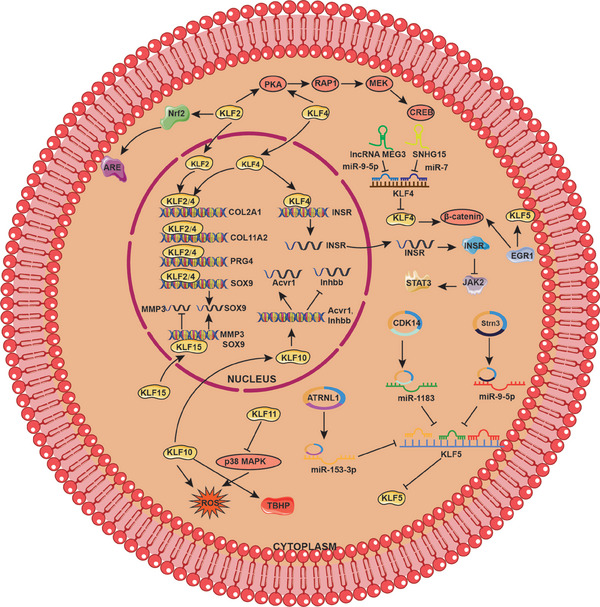
The detailed mechanisms of OA progression through KLFs associated signaling pathways. KLF4 and KLF2 directly bind to cartilage signature genes such as COL2A1, COL11A2, PRG4, and SOX9 to increase their expression. KLF4 and KLF2 are involved in the PKA–RAP1–MEK–CREB signaling axis, ultimately suppressing mediators of inflammation and ECM‐degrading enzymes. KLF4 transcriptionally regulates InsR, inactivating JAK2/STAT3 signaling, thus suppressing apoptosis of IL‐1β‐induced OA chondrocytes. KLF2 activates the Nrf2/ARE signaling pathway to block apoptosis of chondrocytes and matrix degradation. SNHG15 sponges miR‐7 targeting KLF4 to regulate β‐catenin, and LncRNA MEG3 sponges miR‐9‐5p targeting KLF4, thereby inhibiting ECM degradation and cell apoptosis. EGR1 activates KLF5 and β‐catenin signaling to promote cartilage degeneration and hypertrophy. Circ‐Strn3 could sponge miR‐9‐5p targeting KLF5 and CircATRNL1 could sponge miR‐153‐3p targeting KLF5 and circCDK14 could sponge miR‐1183 targeting KLF5, protecting against OA. KLF10 upregulates Acvr1 and downregulates Inhbb to inhibit the proliferation and migration of chondrocytes. KLF10 promotes the TBHP‐induced senescence and ROS production. KLF 11 inhibits the p38 MAPK signaling pathway to suppress oxidative stress and apoptosis. KLF15 activates SOX‐9 expression to promote chondrogenic differentiation of hMSCs. KLF15 could bind to the promoter region of MMP‐3 and inhibit its expression, thereby improving articular cartilage degradation in OA.

MMP‐3 is regarded as one of the most important cartilage‐degrading enzymes that mediate the degradation process of type II collagen and aggrecan. The Yishuo Li group found KLF15 is downregulated in chondrocytes from OA patients, which may perform the protective role in OA chondrocytes. Mechanistically, KLF15 could bind to the promoter region of MMP‐3 and inhibit its expression at the transcriptional level, thereby improving articular cartilage degradation in OA.^133^ Additionally, KLF11 can suppress oxidative stress and apoptosis in OA chondrocytes by inhibiting the p38 MAPK signaling pathway.^132^ KLF10, which is a harmful factor overexpressed in both senescent chondrocytes and cartilage affected by OA,^131^ inhibits the proliferation and migration of chondrocytes via upregulation of Acvr1 and downregulation of Inhbb, significantly accelerating OA progression.^241^ KLF10 deletion attenuates the tert‐butyl hydroperoxide (TBHP)‐induced senescence, blocking ROS production and maintaining mitochondrial homeostasis, thereby protecting cartilage against OA damage^131^ (Figure 5).

### 6.2 Osteoporosis

OP is a common orthopedic disorder, characterized by low bone mineral density (BMD), altered bone microstructure, and increased fracture incidence.^242^, ^243^ The imbalance between OB‐mediated bone formation and OC‐mediated bone resorption is the main contributor to OP.^244^ KLFs participate in the regulation of OB and OC activity.^21^, ^245^

Several studies have demonstrated that KLF10 plays a crucial role in regulating OB differentiation, bone formation, and mineralization processes.^45^, ^209^ KLF10 could not only activate the transcription of the Runx2 gene via directly binding to the promoter and interacting with translated protein,^45^ but also suppress osteoclastogenesis through the repression of the NFATc1 pathway and MEK/ERK signal transduction.^120^ Further, according to quantitative computerized tomography studies, there is strong evidence that KLF10 is associated with volumetric cortical BMD.^246^ Compared with the normal population, the expression of KLF10 is significantly reduced in the skeletal tissues of osteoporotic patients.^247^

KLF4 is reported to modulate bone homeostasis by suppressing both OC and OB differentiation.^112^ However, current studies demonstrated that KLF4 expression is time‐specific and shows corresponding expression levels in different development stages, exerting diverse effects on bone mass. For instance, KLF4 conditional knockout in bone progenitor cells results in decreased OB production in mice, so as to significantly reduce bone mass,^248^ while mice with conditional deletion of KLF4 in OBs exhibit the opposite phenotype with high bone mass caused by the enhancement of bone formation.^112^ Further studies indicated that tumor necrosis factor‐α (TNF‐α) significantly upregulates the expression of SOX5 transcriptionally, which inhibits the expression of OB markers such as Runx2, leading to a suppressive effect on the osteogenic differentiation of adult hMSCs, while KLF4 knockdown can reverse the inhibition.^129^

The local vasculature actively participates in both bone formation and resorption by determining the fate of progenitor cells in the skeletal system. CD31^hi^EMCN^hi^ vessels, located in the metaphysis and endosteum of postnatal long bones, have been proven to couple angiogenesis and osteogenesis.^249^ The Mi Yang group demonstrated that KLF3 could inhibit the expression of JunB and Vegfa, thus repressing the formation of the CD31^hi^Emcn^hi^ endothelium in the bone marrow, ultimately leading to decreased bone mass.^214^ Furthermore, Yang et al.^115^ demonstrated that GC treatment results in impaired bone formation by activating adipogenesis‐associated KLF15/PPARγ2/FoxO3a/Wnt pathway and suppressing the canonical osteogenesis‐related Wnt signaling. Additionally, KLF7 could directly bind to the promoter region of HO‐1 to repress its expression, and thereby promote OC differentiation, ultimately facilitating OP progression.^121^

### 6.3 Osteosarcoma

Primary bone cancers, such as OS, Ewing sarcoma, and chondrosarcoma, are relatively rare but associated with high morbidity and mortality rates.^250^, ^251^ The development of bone cancers is partly ascribed to the metabolic changes caused by signal pathway reprogramming, which are vital to sustaining increased redox, bioenergetic, and biosynthesis demands of a tumor cell.^252^ Recent studies showed that transcription factors play dominant roles in the initiation and advancement of OS, associated with major metabolic pathways.^253^

KLF2 has been shown to have growth‐inhibitory, proapoptotic, and antiangiogenic effects, which are downregulated in malignancies.^254^ Zheng et al.^233^ found that circ‐LRP6 is highly expressed in OS and interacts with LSD1 and EZH2 to mediate their binding to the promoter regions of KLF2, thereby inhibiting KLF2 expression and ultimately promoting OS development. Furthermore, KLF2 is downregulated by SNHG6, a novel type of molecule associated with the progression of multiple cancers, thereby accelerating OS progression.^231^

KLF4 is a crucial regulator of normal cell proliferation and inhibits the proliferation of tumor cells, serving as a suppressor in many cancers. Qi et al.^255^ found that overexpression of KLF4 in OS cells led to the manifestation of traits commonly associated with OS CSCs including heightened sphere‐forming potential, elevated levels of stemness‐associated genes, and increased potential for metastasis. Contrarily, KLF4 knockdown could reduce colony formation in vitro and inhibit tumorigenesis in vivo, further confirming the oncogenic role in OS pathogenesis.^255^ A recent study identified that miR‐135a is downregulated in OS tissue and inhibits cell invasion and expression by directly targeting KLF4.^226^ KLF6 serves as an important tumor suppressor gene frequently downregulated in multiple human cancers.^256^ Zhu et al.^257^ found KLF6 overexpression inhibits the viability, proliferation, and invasion of MG63 cells, and enhances cell apoptosis of the MG63 OS cell line via suppression of bcl‐2 and MMP‐9 and activation of p21.

**FIGURE 6:**
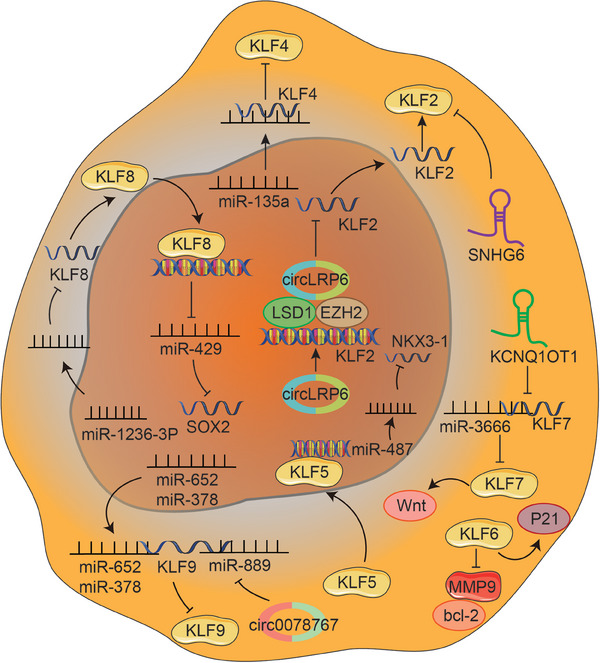
The detailed mechanisms of OS progression through KLFs associated signaling pathways. Circ‐LRP6 interacts with LSD1 and EZH2 to bind to the promoter regions of KLF2, thereby inhibiting KLF2 expression and ultimately promoting OS development. SNHG6 downregulates KLF2 to accelerate OS progression. MiR‐135a targets KLF4 to inhibit cell invasion. KLF5 enhances the expression of miR‐487a that targets NKX3, which significantly facilitates the invasion and metastasis of OS cells. KLF6 suppresses bcl‐2 and MMP‐9 and activates p21 to inhibit proliferation and invasion and enhance cell apoptosis. KCNQ1OT1 targets the miR‐3666 to promote KLF7 expression, activating Wnt/β‐catenin signaling to facilitate OS progression. MiRNA‐1236‐3p downregulates KLF8 to suppress the proliferative ability and induce apoptosis of OS cells. KLF8 binds the promoter region to inhibit the expression of miR‐429, targeting SOX2 to mediate cancer stem cell‐like features. MiR‐378 and miR‐652 target KLF9 to promote the cell proliferation of OS. Circ_0078767 targets miR‐889 to enhance KLF9 expression, ultimately inhibiting OS progression.

MiRNAs serve as the upstream and downstream components of KLFs, widely participating in the occurrence and progression of OS. For instance, miR‐652 negatively regulates KLF9 by directly interacting with its 3′‐UTR, performing a carcinogenic role in OS cells.^227^ Similarly, MiR‐378 also targets KLF9 to promote the cell proliferation of OS.^228^ Circ_0078767 has been demonstrated to enhance KLF9 expression by targeting miR‐889, ultimately inhibiting OS progression.^235^ LncRNA KCNQ1OT1 is highly expressed in human OS tissues, and promotes OS cell proliferation, migration and invasion. Mechanistically, KCNQ1OT1 directly targets the miR‐3666/KLF7 axis and activates Wnt/β‐catenin signaling to facilitate OS progression.^232^ In addition, it is demonstrated that miRNA‐1236‐3p suppresses the proliferative ability and induces apoptosis of OS cells by downregulating KLF8, an important cancer‐promoting modulator.^211^ KLF8 directly binds the promoter region to inhibit the expression of miR‐429, which directly targets SOX2 to mediate CSC‐like features in CD133^+^ OS stem cell‐like cells (OSCs).^230^ Besides, KLF5 enhances the expression of miR‐487a by directly binding to its promoter region, which significantly facilitates the invasion and metastasis of OS cells via targeting NKX3‐1^229^ (Figure 6).

### 6.4 Cancer‐associated bone metastasis

According to research, bone metastasis is the third most common site for cancer metastasis, with around 70% of patients with metastatic prostate and breast cancer developing it.^258^ Bone metastasis is typically classified into three types: osteolytic, osteosclerotic, and mixed. Osteoblastic metastasis is typical for PCa, while breast cancer usually results in osteolytic metastasis.

Recently, increasing evidence indicated that several KLF members are involved in a variety of biological processes during breast cancer progression, including proliferation, migration, invasion, metastasis, and apoptosis.^28^, ^259^ Studies have demonstrated that increased expression of vascular cell adhesion molecule 1 in disseminated breast tumor cells plays a crucial role in attracting pre‐OCs and facilitating their transformation into mature OCs, which ultimately leads to bone metastasis.^260^ Upon bone destruction, TGF‐β is released from the bone matrix and signals to breast cancer, which may induce the expression of KLF10 and KLF11, which can further promote the development of bone metastasis.^155^ Additionally, the Goutham Narla group demonstrated that KLF6‐SV1, a splice variant of KLF6, has been found to be upregulated in hormone‐refractory metastatic PCa. Its upregulation has been linked to the acceleration of PCa progression and metastasis through the modulation of apoptosis, cellular proliferation, and angiogenesis. PCa cells with overexpressed KLF6‐SV1 metastasize more rapidly and disseminate to bone. Inhibition of KLF6‐SV1 leads to spontaneous apoptosis in cultured PCa cell lines and also suppresses tumor growth in mice.^261^

Indeed, bone homeostasis is a dynamic process governed by the balanced activity of OCs, which are responsible for bone resorption, and OBs, which are responsible for bone formation. Disruption of this balance is a hallmark of bone metastasis. Therefore, the regulation of bone metastasis by the KLF family may extend beyond their roles in cancer cell behavior, encompassing significant impacts on bone homeostasis, specifically osteoclastogenesis, and osteoblastogenesis. As mentioned above, various KLF members have been implicated in modulating the differentiation and activity of OCs. Dysregulation of these transcription factors can enhance OC formation, leading to excessive bone resorption. In the setting of cancer metastasis, such enhanced osteoclastic activity can create a favorable environment for cancer cells to invade and thrive within the bone matrix. On the other hand, KLFs also influence OB differentiation and function. Proper regulation of these pathways is essential for maintaining bone integrity. Abnormal expression of KLFs in OBs can impair bone formation, contributing to the imbalance between bone resorption and formation. In the context of osteoblastic metastasis seen in PCa, alterations in KLF expression could potentially enhance OB activity, leading to the characteristic bone lesions associated with this type of metastasis. By influencing both osteoclastogenesis and osteoblastogenesis, KLFs can create a bone microenvironment that either favors bone resorption or bone formation, depending on the type of metastatic cancer. This dual role highlights the importance of KLFs in maintaining skeletal homeostasis and the potential consequences of their dysregulation in the progression of bone metastases. Targeting the specific KLF‐mediated pathways that regulate these processes could offer novel therapeutic strategies to mitigate bone metastasis in cancer patients.

### 6.5 KLFs act as the therapeutic target for treating bone destruction diseases

KLFs modulate bone homeostasis in multiple pathways including transcriptional regulation, energy metabolism as well as epigenetic activity. Aging, inflammation, stress, and other microenvironmental alterations affect the expression or activity of the KLF family, and thus lead to the disorder of downstream gene expression, ultimately causing a series of bone destruction diseases. Given the key role of KLFs in bone‐related diseases (including OA, OP, OS, etc.), the development of drugs targeting KLFs may provide effective therapies for bone destruction diseases. For instance, Quercetin has been shown to decrease the expression of KLF4, which helps protect cells against oxidative damage and can potentially be used to treat oxidative‐related diseases.^262^ Furthermore, Li et al.^263^ reported that adriamycin treatment has been found to increase the expression of KLF4, leading to enhanced metastasis and cancer stemness in OS cells. Fortunately, simvastatin administration can markedly reverse adriamycin‐induced tumorigenesis by downregulating KLF4 expression, providing potential new treatments for OS.^263^ Additionally, Kawata et al.^264^ identified a class I selective HDAC inhibitor moceinostat, which could improve tissue destruction and inflammation in OA by activating KLF4. Thiazolidinedione, a drug for treating type II diabetes, has been proven to KLF11 expression, which may be applied in the treatment of KLF11 silencing‐mediated formation of OS CSCs.^203^, ^265^ These natural products and medicines possess certain curative effects with poor tissue specificity and promiscuity of binding. The regulation of gene expression mediated by miRNAs is owned to virtually all processes of skeleton development and has been shown its biological characteristics in bone destruction diseases, demonstrating the possibilities in the relevant treatment. MiRNA‐based therapies targeting KLFs may provide significant clinical benefits to cancer patients potentially. For instance, BMMSCs‐derived exosomes could enhance OB proliferation via miR‐21‐5p mediated inhibition of KLF3, which may be a potential therapeutic strategy to improve OP.^225^ Further research on the miRNA‐KLF axis is required to fully understand the underlying mechanism, which has the potential to improve targeted therapies for bone destruction diseases.

## 7 CONCLUSIONS AND PERSPECTIVES

Herein, we have concluded the multifaceted roles of KLF on body health and disease, and selectively outline the available evidence regarding the effects of KLFs on the regulation of bone homeostasis, as well as discuss the coupling effects between KLFs and bone remodeling and how metabolic and epigenetic approaches could be employed by targeting KLFs. KLFs participate in the regulation of bone homeostasis, which is a subtle and context‐dependent process involved in nearly all aspects of skeletal development. The complicated roles of the KLF family in the skeletal system coupled with multiple cellular processes such as differentiation, proliferation, migration, and apoptosis, with spatio‐temporal continuity and tissue specificity. Epigenetic determinants including DNA and histone modifications as well as ncRNAs, are the main administrators and implementers of KLF‐mediated gene expression regulation, function in a development‐specific manner. Collectively, these epigenetic determinants, KLF family, and KLF‐targeted downstream genes as well as signaling pathways construct a KLF‐centered regulatory network that enables gene expression to be appropriate for bone physiological processes.

Abnormal KLF expression caused by various factors will affect the expression of downstream molecules, thus mediating the occurrence of pathological changes. In light of the crucial function of the KLF family members in a variety of diseases, potential therapies for targeting these molecules have become promising therapeutic strategies in the treatment of many diseases. The investigation of KLF family offers a fresh perspective for gaining a comprehensive understanding of multiple system biology. Furthermore, by examining the action of KLFs in bone cells, we can enhance our comprehension of bone development and diseases, as well as contribute to the advancement of valuable novel treatment approaches for bone‐related disorders.

However, several major challenges are identifying specific modulation measures on KLFs, which enable to display the close communication among varying cells in the dynamic balance of microenvironment in bone homeostasis. In conclusion, further studies on the effector mechanisms of the KLF signaling pathway and its multiple modifications at different levels will provide novel insights into developing effective therapeutic targets for treating multiple disorders in the skeletal system.

## AUTHOR CONTRIBUTIONS

Tingwen Xiang and Chuan Yang were major contributors in writing the manuscript and creating all the figures and tables. Tingwen Xiang, Chuan Yang, and Zihan Deng performed literature search. Dong Sun, Fei Luo, and Yueqi Chen made substantial contributions to the design of the manuscript and revised it critically for important intellectual content. All authors have read and approved the final version of this manuscript.

## CONFLICT OF INTEREST STATEMENT

The authors declare that they have no known competing financial interests or personal relationships that could have appeared to influence the work reported in this paper.

## ETHICS STATEMENT

Not applicable.

## Data Availability

All data are available from the corresponding authors upon reasonable request.
